# A Novel Universal Detection Agent for Time-Gated Luminescence Bioimaging

**DOI:** 10.1038/srep27564

**Published:** 2016-06-10

**Authors:** Nima Sayyadi, Andrew Care, Russell E. Connally, Andrew C. Try, Peter L. Bergquist, Anwar Sunna

**Affiliations:** 1 Macquarie University, Department of Chemistry and Biomolecular Sciences, Sydney, NSW 2109, Australia; 2 Macquarie University, ARC Centre of Excellence for Nanoscale BioPhotonics, Sydney, NSW 2109, Australia; 3 Macquarie University, Department of Physics and Astronomy, Sydney, NSW 2109, Australia; 4 University of Auckland, Department of Molecular Medicine and Pathology, Auckland, 92019, New Zealand

## Abstract

Luminescent lanthanide chelates have been used to label antibodies in time-gated luminescence (TGL) bioimaging. However, it is a challenging task to label directly an antibody with lanthanide-binding ligands and achieve control of the target ligand/protein ratios whilst ensuring that affinity and avidity of the antibody remain uncompromised. We report the development of a new indirect detection reagent to label antibodies with detectable luminescence that circumvents this problem by labelling available lysine residues in the linker portion of the recombinant fusion protein Linker-Protein G (LPG). Succinimide-activated lanthanide chelating ligands were attached to lysine residues in LPG and Protein G (without Linker) and the resulting Luminescence-Activating (LA-) conjugates were compared for total incorporation and conjugation efficiency. A higher and more efficient incorporation of ligands at three different molar ratios was observed for LPG and this effect was attributed to the presence of eight readily available lysine residues in the linker region of LPG. These Luminescence-Activating (LA-) complexes were subsequently shown to impart luminescence (upon formation of europium(III) complexes) to cell-specific antibodies within seconds and without the need for any complicated bioconjugation procedures. The potential of this technology was demonstrated by direct labelling of *Giardia* cysts and *Cryptosporidium* oocysts in TGL bioimaging.

Lanthanide (e.g., Eu^3+^, Tb^3+^) ions are of growing interest as luminescent probes for time-gated luminescence (TGL) bioimaging^1^. The outstanding luminescent properties of lanthanide ions are characterised by their sharp emission profiles (<10 nm width), large Stokes shifts (>150 nm) and long (millisecond) excited-state lifetimes. These features, in conjunction with pulsed excitation and time-gated measurements, allow temporal discrimination against fast decaying (nanosecond) autofluorescence and scattered excitation light^2^. TGL microscopy has been used successfully to visualise biomolecules and cells in autofluorescent environments^3^ by exploiting the long luminescent lifetimes of lanthanide ions.

Trivalent lanthanide ions (Ln^3+^) have intrinsically low absorption cross-sections, thus direct excitation yields only low levels of luminescence. As a result, lanthanide ions need to be excited indirectly through a method known as sensitisation, where a lanthanide ion is chelated by an organic ligand containing a chromophore that acts as an antenna to sensitise the absorption of light and transfer of excitation energy to the chelated ion, resulting in higher luminescence and extended emission lifetimes^4^. This construction is referred to as a lanthanide chelate. Ligands capable of lanthanide binding can be attached to a biomolecule (e.g., antibodies and nucleic acids) *via* a cross-linking group.

A number of highly luminescent tetradentate bis *β*-diketonate-Eu^3+^ chelates have been used to label antibodies in TGL bioimaging (e.g., 4,4′-bis(1″,1″,1″,2″,2″,3″,3″-heptafluoro-4″,6″-hexanedion-6″-yl)-chlorosulfo-*o*-terphenyl (BHHCT)^5^ and 4,4-bis-(1″,1″,1″,2″,2″,3″,3″-heptafluoro-4″,6″-hexanedion-6″-yl) sulfonylaminopropyl-ester-*N*-succinimide-ester-*o*-terphenyl (BHHST)^6^, but the difficulties associated with the direct incorporation of lanthanide chelates onto antibodies (*via* the initial attachment of the lanthanide ion-binding ligands) are well documented. For example, BHHCT is known to cause antibody inactivation or precipitation due to poor aqueous solubility, over-conjugation of ligand to the antibody, and variations in antibody reactivity and sensitivity^6^,^7^. Hence, direct antibody modification is often inefficient and requires time-consuming optimisation, a process unique to a given antibody. Indirect methods that deliver sufficient luminescent signal with retention of antibody function have been established. For example, Connally *et al*.^6^ applied BHHST-labelled secondary antibodies for the indirect detection of *Giardia* with TGL microscopy but direct modification of a secondary antibody makes it susceptible to inactivation in the same fashion as directly labelling a primary antibody. Alternatively, lanthanide-labelled streptavidin has been used as an indirect detection reagent to label biotinylated secondary antibodies with detectable luminescence for TGL bioimaging^8^. Even so, this method requires the specific modification of antibodies with biotin and relies on the biotin-streptavidin binding interaction, which can be difficult to control. Streptavidin conjugated proteins have also the tendency of binding non-specifically to biotinylated proteins in mammalian cells resulting in unpredictable background problems^9^,^10^,^11^. Additional chelates can be loaded onto a carrier molecule that tolerates a high degree of labelling and can be chemically cross-linked to a detection reagent to maximise luminescence further. For example, streptavidin typically is conjugated to bovine serum albumin (BSA), and then the conjugate is labelled with lanthanide chelates prior to use as an indirect detection reagent in TGL bioimaging^1^,^12^,^13^,^14^.

Another potential universal detection reagent is the recombinant fusion protein, Linker-Protein G (LPG), the subject of this report. LPG contains two functionally distinct regions; (a) a peptide linker sequence which has specific binding affinity towards silica-containing materials, and (b) *Streptococcus* Protein G′ which has specific binding affinity towards antibodies^15^. LPG has been used as an anchor point for the oriented immobilisation of antibodies onto silica-containing materials without the need for complex surface chemical modification^15^,^16^,^17^,^18^,^19^. In addition, the linker region of LPG presents itself as a prospective lanthanide carrier. It contains a number of accessible lysine residues whose terminal amino groups provide binding sites for succinimide-activated ligands capable of binding europium(III), ultimately allowing the addition of multiple luminophores with minimal effect on the antibody-binding ability of the Protein G region.

4,4′-Bis(1″,1″,1″,2″,2″,3″,3″-heptafluoro-4″,6″-hexanedion-6″-yl)sulfo-*o*-terphenyl-tetraethylene glycol-*N*-hydroxysuccinimide (BHHTEGST)^20^ is a newly developed tetradentate bis *β*-diketone-ligand derived from BHHST^6^ and BHHCT^21^, capable of binding europium(III). It has good aqueous solubility, excellent luminescent output (when complexed with europium(III)) and a mild succinimide reactive group that facilitates covalent attachment to biomolecules that contain lysine residues. BHHTEGST also possesses an extended tetraethylene glycol tether that projects its hydrophobic europium(III) binding moiety (BHHCT) away from the modified protein to prevent adverse interactions and enhance conjugate solubility.

In this communication, we show that LPG labelled with BHHTEGST serves as an effective indirect detection reagent to impart luminescence rapidly to antibodies for TGL bioimaging. We demonstrate also that the presence of the linker region significantly increases luminescent output, enabling rapid and simple visualisation of *Giardia* cysts and *Cryptosporidium* oocysts under time-gated conditions.

## Results

### Conjugation of PG (without Linker) and LPG with BHHTEGST

PG and LPG must be able to withstand extensive conjugation with the BHHTEGST ligand and retain their capacity to bind antibodies for use as universal detection reagents in TGL bioimaging. Thus, each protein underwent conjugation reactions at different BHHTEGST:Lysine ratios, yielding a total of three Luminescence-Activating (LA-) conjugates (Table 1), which will be referred to in this manuscript as LA-PG_HIGH, MID or LOW_ and LA-LPG_HIGH, MID or LOW_. The average number of ligands attached to LA-PG or LA-LPG at each conjugation ratio was then semi-quantified as described in the Materials and Methods section and the results are summarised in Table 1.

The BHHTEGST ligand is amine reactive and attaches covalently to lysine residues in target proteins. PG contains 19 lysine residues whereas LPG has 27 due to the presence of an additional 8 lysines located in the linker region. Therefore it was expected that LPG would be conjugated with a higher number of ligands at each BHHTEGST:Lysine ratio tested. As shown in Table 1, in conjugations performed at the lowest BHHTEGST:Lysine ratio, PG was modified with only an average of 4.3 ligands whereas LPG had 12.3. This difference (8 ligands) correlates with the additional eight ligands present on the linker region of LPG. A similar difference (7.1 ligands) in the number of attached ligands was observed at the mid BHHTEGST:Lysine ratio, in which PG and LPG contained on average 9.7 and 16.8 ligands, respectively. However, this difference was considerably smaller (2.5 ligands) at the highest BHHTEGST:Lysine ratio tested, which resulted in PG and LPG complexes that contained on average 15.5 and 18.0 BHHTEGST ligands, respectively. These results show that better and more consistent conjugation efficiencies were achieved with LPG (45.7–66.7%) compared to PG (22.8–81.6%) across the range of BHHTEGST:Lysine ratios tested. More importantly, the results of the conjugation studies at low and medium levels suggest that the incorporation of the additional BHHTEGST ligands (8 and 7.1, respectively) takes place preferentially on the linker region of LPG, remote from the antibody-binding region.

### Using lanthanide-activated complexes to label *Giardia* and *Cryptosporidium*

**Figure 1:**
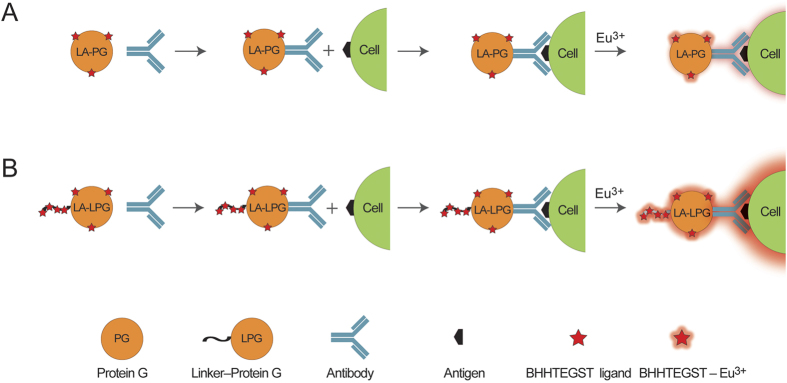
Illustration showing the luminescent labelling of cells using LA-PG- and LA-LPG-coupled antibodies. Coupling of LA-PG (**A**) or LA-LPG (**B**) to an antibody results in a “LA-PG or LA-LPG–Antibody” conjugate that can be used to label a target cell with luminescence upon exposure to Eu^3+^. The red stars represent an unspecific number of BHHTEGST ligands incorporated onto the LA-PG (**A**) or LA-LPG (**B**) biomolecule.

**Figure 2:**
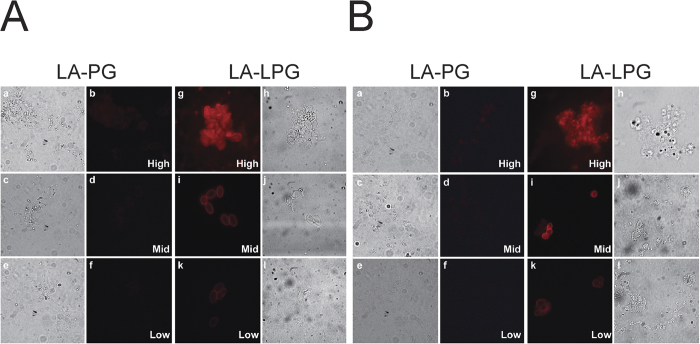
Luminescent labelling of (**A**) *Giardia* cysts and (**B**) *Cryptosporidium* oocysts using (a–f) LA-PG_HIGH, MID, or LOW_ or (g–l) LA-LPG_HIGH, MID, or LOW_ -coupled antibodies. (a, c, e and h, j, l) Bright-field microscopy. (b, d, f and g, i, k) TGL microscopy. Exposure time for TGL microscopy was identical for all samples; as a result the brightness of the images are relative to each other.

We investigated whether the lanthanide-activated complexes could act as universal detection reagents to impart detectable luminescence to primary antibodies without compromising their function and which conjugation ratio was the most effective. Using the strategy illustrated in Fig. 1, each of the modified conjugates from the previous section was coupled to cell-specific antibodies for the luminescent labelling of *Giardia* cysts (Fig. 2A) and *Cryptosporidium* oocysts (Fig. 2B), following exposure of slides to a europium(III) solution. The labelled cells were visualised by TGL microscopy using a Gated Auto-synchronous Luminescence Detector (GALD)^3^ and the SNRs from the raw digital images were calculated using ImageJ software (Table 2).

Of all the complexes studied (Fig. 2), LA-LPG_MID_ images showed high definition and SNRs of 76 for *Giardia* and 71 for *Cryptosporidium* (Table 2). In contrast, the cells labelled with the LA-PG_MID_ complex exhibited low luminescence and poor resolution. Accordingly, the SNRs in these images were significantly lower for both cell-types (*Giardia* = 7; *Cryptosporidium* = 16). The LA-LPG_MID_ complex provided more than a 10-fold enhancement in the SNRs of labelled *Giardia* luminescence and more than a 4-fold enhancement for *Cryptosporidium* when compared to LA-PG_MID_. Cells labelled with LA-LPG_HIGH_ displayed similar SNRs (*Giardia* = 72; *Cryptosporidium* = 76) to those shown by LA-LPG_MID_. However, the cells appeared oversaturated with poorly-defined boundaries. The LA-PG_HIGH_ control generated low SNRs (*Giardia* = 16; *Cryptosporidium* = 21) and produced images that showed weak luminescence and low resolution. In comparison to LA-PG_HIGH_, the LA-LPG_HIGH_ complex improved the SNRs of the labelled *Giardia* and *Cryptosporidium* by more than 4-fold and 3-fold, respectively. Low SNRs were obtained from cells labelled with the LA-LPG_LOW_ complex (*Giardia* = 20; *Cryptosporidium* = 25) and the cells showed very weak luminescent output and poor definition with the weakest SNRs produced by cells labelled with LA-PG_LOW_ (*Giardia* = 6; *Cryptosporidium* = 10). In these images the cells were difficult to detect and identify. When compared to LA-PG_LOW_, the LA-LPG_LOW_ complex amplified SNRs by more than 3-fold and 2-fold for labelled *Giardia* and *Cryptosporidium*, respectively.

These results indicated that all of the modified complexes can bind antibodies and render them luminescent, but with greatly varied efficacy. The cells labelled with LA-LPG were significantly brighter in label intensity than LA-PG (irrespective of the conjugation concentration) an observation that can be attributed to the higher BHHTEGST content of the LA-LPG conjugates (see Table 1) and hence, europium(III) chelates. However, LA-LPG_HIGH_ possesses the highest BHHTEGST content and produced images in which cells were highly luminescent but poorly defined. This lack of definition could possibly be due to a reduction in cell targeting efficiency. The LA-LPG_MID_ conjugate provided clear, high-contrast and definition images, making it the best detection reagent under these conditions for the attachment to the antibodies tested. Therefore, all subsequent cell-labelling experiments were performed using LA-LPG_MID_-coupled antibodies.

### Detection of cells in autofluorescent environments

**Figure 3:**
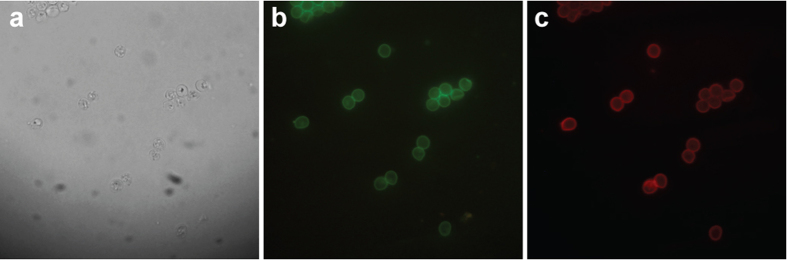
*Cryptosporidium* oocysts labelled with “LA-LPG_MID_ + CRY104-FITC” complex. (**a**) Bright field (**b**) FITC channel (**c**) TGL conditions.

Lanthanide chelates have long luminescence lifetimes that enable temporal discrimination of shorter-lived autofluorescence, unlike conventional fluorophores. We tested whether LA-LPG_MID_-coupled antibodies in conjunction with TGL microscopy can be used for background-free detection of cells in autofluorescent environments (Fig. 3). In an initial control experiment, the LA-LPG_MID_ complex was coupled to a FITC-conjugated antibody (CRY104-FITC) specific for the cell walls of *Cryptosporidium* oocysts. Cells were then labelled and visualised by either fluorescence microscopy or TGL microscopy. Cells observed in the fluorescent FITC channel displayed bright green fluorescence and good definition (Fig. 3b).

**Figure 4:**
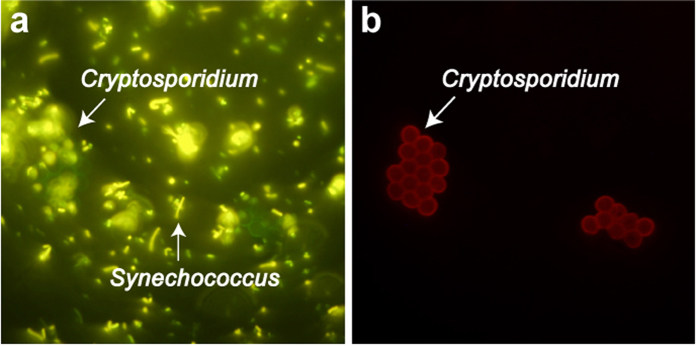
A sample of *Cryptosporidium* oocysts was spiked with autofluorescent *Synechococcus* cells and then labelled with “LA-LPG_MID_ + CRY104-FITC” complex. (**a**) Bright field (**b**) FITC channel (**c**) TGL conditions.

Under time-gated conditions the cells emitted red luminescence and maintained their definition (Fig. 3c). These results indicated that LA-LPG_MID_ can be coupled easily to a different fluorophore-conjugated antibody and applied as a dual-immuno-labelling technique based on emission lifetimes. Consequently, we used this methodology to compare the efficiency of a conventional fluorophore (FITC) and our lanthanide-carrier molecule (LA-LPG_MID_) to label cells in an autofluorescent environment (Fig. 4). *Cryptosporidium* oocysts were mixed with a sample of autofluorescent *Synechococcus* cells and then labelled with the “LA-LPG_MID_ + CRY104-FITC” complex (that was used to obtain the images in Fig. 3). The *Cryptosporidium* oocysts were fluorescent green in the FITC channel as expected, but difficult to discriminate against background autofluorescence provided by the *Synechococcus* cells (Fig. 4a). Switching to time-gated microscopy suppressed the background autofluorescence and generated high-contrast images in which cells exhibited red luminescence and were identified readily against a fluorescence-free background (Fig. 4b). These results show that the LA-LPG_MID_ complex in combination with TGL microscopy can be used successfully for the sensitive detection of cell targets in the presence of background autofluorescence.

## Discussion

We labelled the lysine residues within PG and LPG with three different molar ratios of BHHTEGST and observed different levels of incorporation (Table 1). Overall, LA-LPG had a greater number of attached BHHTEGST ligands than LA-PG at each ratio tested. The conjugation efficiency of LA-PG decreased almost proportionality to the molar ratio of BHHTEGST, whereas LA-LPG underwent less obvious reductions in conjugation efficiency as the molar ratio of BHHTEGST was lowered. Such differences may be explained by variations in lysine content and accessibility of lysine residues on the linker and Protein G regions. The linker accounts for ~30% of the lysine residues on LPG and can be modified with up to 8 amine-reactive ligands. The intrinsic structural disorder of the linker^19^ may allow it to undergo conformational changes that enable interaction between its lysine residues and BHHTEGST, such that ligand attachment in this linker region is favoured at lower molarities in comparison with attachment at lysines residues on the Protein G component. In contrast, PG has a highly stable and ordered structure. PG also retains its activity at extreme temperatures and across a wide range of pH values. High concentrations of denaturant are required to unfold the protein^22^. The IgG-binding domains of PG have structural homology with the rigid and inflexible protein, ubiquitin^23^. Thus, unlike the linker, the PG region cannot extensively alter its conformation to interact readily with BHHTEGST, preventing high levels of incorporation at lower molarities. Further analysis of the amino acid sequence of LPG using a neural network predictor of natural disordered regions (PONDR, http://pondr.com) was performed (results not shown). It identified the ordered structure of the Fc-binding domains within PG and confirmed the disordered structure of the linker region. We compared the relative SNRs of *Giardia* cysts and *Cryptosporidium* oocysts that were labelled with either LA-PG or LA-LPG complexes (Table 2) to determine which LA-complexes were the most compatible for TGL bioimaging. We found that cells labelled with LA-PG complexes exhibited weak luminescence that was unacceptable for TGL microscopy. Conversely, cells labelled with LA-LPG complexes displayed up to 10-fold higher SNRs. This result shows that the linker region can act as an effective lanthanide-carrier portion of the LPG molecule that is capable of bearing additional chelates to increase the luminescent output of LPG for TGL bioimaging applications.

LA-LPG_MID_ provided bright, high-contrast and high-definition images, making it the best LA-complex for indirect detection in TGL bioimaging. It contains approximately seventeen BHHTEGST ligands (Table 1), if the eight lysine residues on the linker are preferentially modified (as discussed above) then the remaining nine BHHTEGST ligands are likely to be located on the PG region. Markela *et al*.^24^ reported that Protein G retained optimal affinity and binding specificity for IgGs when labelled with up to nine europium chelates. The results with the LA-LPG_MID_ complex highlight the linker’s ability to impart high luminescence to a detection reagent without affecting its binding avidity and specificity.

A loss in binding specificity generally occurs when the detection reagent is over-modified^1^. In this study, the LA-LPG_HIGH_ complex had the highest BHHTEGST ligand content and produced cellular images with high SNRs, but the labelled cells were poorly defined and a large amount of non-specific binding was observed. We propose that this lack of specificity is due to the over-modification of LA-LPG_HIGH_ reducing its affinity towards antibodies. The over-modification of antibody-binding proteins (ABPs) with lanthanide chelates has been observed to reduce their antibody-binding affinity^24^,^25^,^26^,^27^. For example, the attachment of chelates to just 15% of the available lysine residues on Protein A results in ~50% loss in its binding affinity^25^. Furthermore, the attachment of different chelate types can vary the affinity of ABPs towards antibodies from different species^27^.

**Figure 5:**
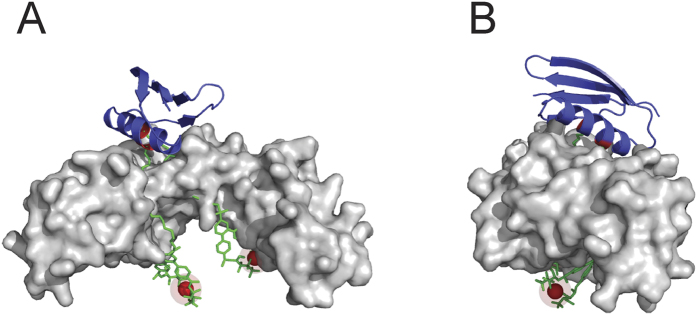
Protein structure models of an IgG-binding domain (C2) from Protein G (blue) in complex with the Fc-region of human IgG (grey). (**A**) The two lysine residues located on the *α*-helix of the IgG-binding domain (marked in red) have each been modified with the BHHTEGST (green)-Eu^3+^ (red ball) complex, resulting in unfavorable steric interactions in these models. (**B**) 90° view.

The BHHTEGST ligand is a molecule that becomes covalently attached to proteins *via* lysine amino groups. The truncated variant of PG^28^ used here includes two domains with high affinity towards the Fc region of IgGs^29^. Both domains consist of a four-stranded *β*-sheet crossed diagonally by a single *α*-helix. X-ray crystallography^23^ and site-directed mutagenesis studies^30^ have identified two lysine residues at the centre of the *α*-helix that are integral to the IgG-binding interaction. Therefore, preservation of the four lysine residues on the *α*-helices of the two IgG-binding domains of PG is essential for optimum binding affinity and specificity. We used a protein model that depicts an IgG-binding domain (i.e., the C2 domain) from Protein G in complex with the Fc region of human IgG (PDB - 1FCC; Fig. 5) to investigate further the potential impact of BHHTEGST incorporation into PG or LPG. In this model, BHHTEGST-Eu^3+^ chelates were attached to the two lysines present on the *α*-helix of the IgG-binding domain that participate in antibody-binding. Both of the relatively large BHHTEGST-Eu^3+^ chelates were shown to project from this domain through the entire Fc region of the bound human IgG. Therefore, any modification of these residues with BHHTEGST could sterically hinder the binding interactions between PG and antibodies. With LPG the accessibility and preferential modification of lysine residues within the linker region positions the BHHTEGST ligands away from the antibody-binding region (PG), thus reducing the potential modification of residues that mediate antibody-binding.

TGL bioimaging has relied on lanthanide-labelled streptavidin as an indirect detection reagent to deliver sufficient luminescence to antibodies without impeding their biological function^8^. This methodology requires the modification of secondary antibodies with biotin and cell-labelling procedures can take up to 24 h^1^,^8^,^12^,^13^,^14^. As an alternative, ABPs such as Protein G and Protein A, rapidly bind with high affinity and specificity to a broad spectrum of IgG species and subclasses without influencing their capacity to recognise and bind antigens^29^. ABPs labelled with lanthanide chelates have been used in the past as detection reagents for indirect time-resolved fluoroimmunoassays (TR-FIA)^24^,^31^,^32^. We have shown that LA-PG and LA-LPG complexes can be used as indirect detection reagents for TGL bioimaging and impart luminescence to antibodies within seconds and without the need for antibody-modification. It should be noted that Protein G labelled with lanthanide chelates are commercially available as an indirect detection reagent for TR-FIA in the dissociative enhancement format (DELFIA)^24^. In this method, a protein is labelled with non-photoactive chelates that bind lanthanide ions. The ions are then dissociated and released into an enhancement solution in which they become luminescent and can be detected. Unfortunately this method is incompatible with TGL microscopy because spatial resolution would be lost in a dissociation-enhanced format. For this reason, TGL microscopy requires detection reagents to contain intrinsically luminescent lanthanide chelates (e.g., tetradentate bis *β*-diketonate-Eu^3+^ chelates) to label bio-targets effectively^1^,^5^,^8^,^12^,^13^,^14^,^33^. Although ABPs labelled with conventional fluorophores (e.g., FITC and Alexa Fluor) are available commercially for use as indirect detection reagents in fluorescence microscopy, we are not aware of any reports in which ABPs have been labelled with intrinsically luminescent chelates for TGL microscopy.

Typically, indirect detection reagents are cross-linked to carrier molecules and the resulting conjugate (e.g., SA-BSA) is then labelled with luminescent chelates^1^,^5^,^12^,^13^,^14^,^33^ allowing higher luminescence for TGL bioimaging to be achieved. In some cases detection reagents have been protein engineered to substitute a small number of surface residues with lysines to improve chelate incorporation^34^. Here we investigated whether the linker region of LPG could serve as a carrier segment for BHHTEGST ligands and subsequent europium(III) chelation. Conventional carrier molecules often reduce the specificity of the detection reagent to which they are cross-linked^35^. For example, Ius *et al*.^25^ described a 55% loss in the IgG-binding affinity of Protein A after it was cross-linked to lanthanide-labelled BSA. However in the case of LPG, the detection reagent (PG) is genetically-fused to the carrier molecule (linker) which circumvents protein cross-linking. To our knowledge, this property is unique to the LPG fusion protein and has not been reported for other detection reagents used for TGL microscopy. Traditional carrier molecules also tend to have large masses and varying stabilities that make it difficult to maintain their solubility after conjugation. The linker overcomes these complications due to its relatively small molecular mass (~10 kDa) and high hydrophilicity. Accordingly, LPG is theoretically 41% more soluble than PG (based on their GRAVY index^36^) which may allow more flexible conjugation procedures and higher labelling ratios with less soluble lanthanide binding ligands (e.g., BHHCT).

LA-LPG represents a novel indirect universal detection reagent that rapidly labels virtually any antibody capable of binding to Protein G with luminescence within seconds. This is property is due to the two different regions of LA-LPG. The Protein G region has the ability to bind to a broad range of mammalian IgGs with high affinity and binding specificity, allowing for an easy interchange of IgGs for different targets. The linker region of LA-LPG functions as a genetically-fused carrier molecule that can be loaded with lanthanide chelates to maximise luminescence for TGL bioimaging. LA-LPG can be used to directly label antibodies for several applications, e.g., bioimaging, cell labelling and detection, and flow cytometry.

## Methods

### Source of materials

BHHTEGST was synthesised as described previously^20^. Recombinant LPG was produced in *E. coli* and purified by ion exchange chromatography as described previously^15^. Recombinant PG was purchased from Sigma-Aldrich (Australia).

### Cells and antibodies

*Cryptosporidium* oocysts and *Giardia* cysts, *Cryptosporidium* monoclonal antibodies CRY104 and CRY104-conjugated to fluorescein isothiocyanate (CRY104-FITC) specific to the walls of *Cryptosporidium* oocysts, and the *Giardia* monoclonal antibody G203 specific to the walls of *Giardia* cysts were purchased from BTF Pty Ltd (Sydney, Australia). Cells from the cyanobacterium *Synechococcus* were kindly provided by Dr Deepa Varkey (Macquarie University).

### Conjugation of proteins with BHHTEGST

BHHTEGST contains an *N*-hydroxysuccinimide ester that enables its attachment to proteins *via* the amino group of lysine residues. Assuming that all of the lysine residues were accessible for reaction, each protein (PG without linker, 19 residues and LPG, 27 residues) was conjugated with three different molar ratios of the BHHTEGST ligand per lysine residue. This is referred to here as the BHHTEGST:Lysine ratio. All conjugation reactions were performed in triplicate. In the conjugation reaction, 100 *μ*g protein was exchanged into 100 mM NaHCO3, pH 8.5 and then mixed with different molar excesses of BHHTEGST. After incubation for 1 h at 37 °C the reaction mixtures were passed through a Sephadex G-25 column in water to remove excess unconjugated BHHTEGST. The fractions corresponding to labelled conjugates were detected using a spectrophotometer (280 and 320 nm), combined and concentrated to 100 *μ*L using an Amicon Ultra centrifugal filter (10 kDa cut-off, Millipore).

### Relative quantification of BHHTEGST ligands attached to proteins

The UV spectrum of BHHTEGST exhibits a maximum absorption at 335 nm and partial absorption at 280 nm, which overlaps with that from both proteins (PG and LPG). Thus, to account for the partial absorption of BHHTEGST in conjugated proteins, molar extinction coefficients for BHHTEGST were calculated from standard curves at both 320 nm and 280 nm as described previously^20^. The concentration of BHHTEGST was calculated from the absorbance of conjugates at 320 nm and the extinction coefficient of BHHTEGST at 320 nm. Then partial absorption of BHHTEGST at 280 nm was identified from its standard curve (at 280 nm). The final protein concentration was obtained after subtracting the absorbance of BHHTEGST from the absorbance of conjugated protein at 280 nm. The average number of BHHTEGST molecules per protein was then determined by dividing the BHHTEGST concentration by the protein concentration.

### Standard cell-labelling procedure

To couple the conjugates to cell-specific antibodies, each conjugate (~2 *μ*g) was mixed with antibody (2 *μ*g) in 100 mM phosphate-buffered saline, pH 7.4 to a final volume of 10 *μ*L and incubated for 30 s at room temperature. To label cells, 5 *μ*L of the conjugate-coupled antibodies were loaded onto a microscope slide containing fixed target cells and incubated for 1 min at room temperature. Cell fixation was carried out by gently drying a 10 *μ*L cell sample on a slide with a hot plate at 40 °C for 20 s. The slide was gently rinsed with MilliQ water. Then 3 *μ*L of 22 mM europium(III) chloride (EuCl_3_) was added to the slide and allowed to react with the ligands for 30 s to form the luminescent chelates. The labelled cells were examined using bright-field, fluorescence and TGL microscopy.

### Microscopy and image analysis

All microscopy and imaging was performed on an Olympus BX51 fluorescence microscope with a UPLSAPO 100 X oil immersion objective lens. Colour images were captured by an Olympus 12.8 megapixel DP72 camera with a sensor resolution of 4140 × 3096 and stored in the TIFF format. To view TGL, a previously described Gated Auto-synchronous Luminescence Detector (GALD) was inserted into the DIC slot of the microscope nosepiece^3^. Cells labelled with luminescence were detected using the GALD without any fluorescence filter and exposure times of 3 s. Fluorescence imaging was carried out using a 100 W mercury arc lamp and a FITC filter set with 200 ms exposure times. The reduction in exposure time from 3 s to 200 ms was required to attain comparable image/pixel brightness. All images in this work are as originally sampled and without post processing.

The luminescent labelling of cells was quantified by analysing the raw digital images with the freeware ImageJ downloaded from the NIH website (http://rsb.info.nih.gov/ij). In each image, a line-plot was placed across the cells. The 8-bit value of the pixels in the red channel was used to attain the peak intensity and was defined as the ‘signal’. The mean intensity from a selected section in the darkest region (cell free) was defined as the ‘noise’. These values were then used to calculate signal-to-noise ratios (SNR). SNRs were normalised to 1 by dividing the ‘signal’ value by the ‘noise’ value (an example is provided in Supplementary Information).

## Additional Information

**How to cite this article**: Sayyadi, N. *et al*. A Novel Universal Detection Agent for Time-Gated Luminescence Bioimaging. *Sci. Rep.* **6**, 27564; doi: 10.1038/srep27564 (2016).

## Supplementary Material

Supplementary Information

## Figures and Tables

**Table 1:** Semi-quantification of the number of BHHTEGST ligands attached to PG and LPG after conjugation reactions were performed in triplicate with BHHTEGST:Lysine ratios between 1.6-0.4.

Conjugation ratio	Relative number of ligands				BHHTEGST:Lysine ratio^a^	
	LA-PG	CE (%)^b^	LA-LPG	CE (%)	LA-PG	LA-LPG
High	15.5 ± 1.4	81.6 ± 7.3	18.0 ± 0.7	66.7 ± 0.7	1.5	1.6
Mid	9.7 ± 2.8	51.2 ± 14.8	16.8 ± 0.6	62.1 ± 0.6	0.7	0.8
Low	4.3 ± 0.6	22.8 ± 3.1	12.3 ± 0.2	45.7 ± 0.2	0.4	0.4

^a^BHHTEGST:Lysine ratio is the number of moles of BHHTEGST added per lysine residue in each protein. ^b^CE = Conjugation Efficiency (%) of total lysine residues modified with BHHTEGST).

**Table 2:** Signal to noise ratios (SNRs) for *Giardia* cysts (Fig. 2A) and *Cryptosporidium* oocysts (Fig. 2B) labelled with each of the LA-PG and LA-LPG complexes.

Conjugation ratio	Signal-to-Noise ratios					
	*Giardia* cysts			*Cryptosporidium* oocysts		
	LA-PG	LA-LPG	Fold diff.^*a*^	LA-PG	LA-LPG	Fold diff.
High	16	72	4.5	21	76	3.6
Mid	7	76	10.9	16	71	4.4
Low	6	20	3.3	10	25	2.5

SNR analysis of raw image data was performed using ImageJ software. ^*a*^Fold diff. = Fold difference.
