# Supplementary material for: A Novel Universal Detection Agent for Time-Gated Luminescence Bioimaging
Source: Sci Rep. 2016 Jun 10;6:27564. doi: 10.1038/srep27564 (PMC4901361; doi:10.1038/srep27564)
Supplement: Supplementary Information [file srep27564-s1.pdf]

Supplementary Information

A Novel Universal Detection Agent for Time-Gated Luminescence Bioimaging

Nima Sayyadi [a,b] †, Andrew Care [a,b]†, Russell E. Connally [c], Andrew C. Try [a], Peter L. Bergquist[a,d] and Anwar Sunna [a*]

† These authors contributed equally to this work.

[a] Department of Chemistry and Biomolecular Sciences, Macquarie University, Sydney.

[b] ARC Centre of Excellence for Nanoscale BioPhotonics, Macquarie University, Sydney.

[c] Department of Physics and Astronomy, Macquarie University, Sydney.

[d] Department of Molecular Medicine & Pathology, Medical School, University of Auckland, Auckland, New Zealand.

[*] Corresponding author email: anwar.sunna@mq.edu.au

Supplementary Materials

Image Analysis

The luminescent labelling of cells was quantified by analysing the raw digital images with ImageJ software (http://rsb.info.nih.gov/ij). An example of this method is provided below (Fig. S1 and Table S1).

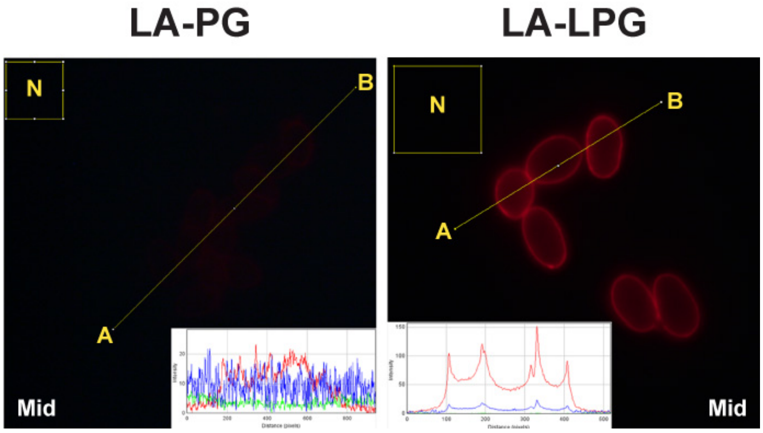

Fig. S1. Raw image analysis of the luminescent output from *Giardia* cysts labelled with either LA-PG_{MID} or LA-LPG_{MID} performed using ImageJ software. Line profiles of the 8-bit pixel intensities in the red, green and blue channels of each line plot (from A to B) are inset. (N) Selected region defined as background or 'Noise'.

Table S1. Calculation of normalised signal-of-noise ratios from *Giardia* cysts labelled with either LA-PG_{MID} or LA-LPG_{MID} (Fig. S1) performed using ImageJ software.

	**LA-PG**	**LA-LPG**
Peak signal (S) from A-B line plot[a]	23	152
Noise (N)[b]	3.3	2
Signal-to-Noise Ratio (SNR)	23:3.3	152:2
**Normalised[c] SNR**	**7:1**	**76:1**

[a]Peak signal intensity observed of the pixels in the red channel; the maximum possible value is 255.

[b]Mean intensity from a selected section in the darkest region (cell free) of the image.

[c]SNRs were normalised to 1 by dividing the ‘signal’ (S) value by the ‘noise’ (N) value.

Relative quantification of BHHTEGST ligands attached to proteins

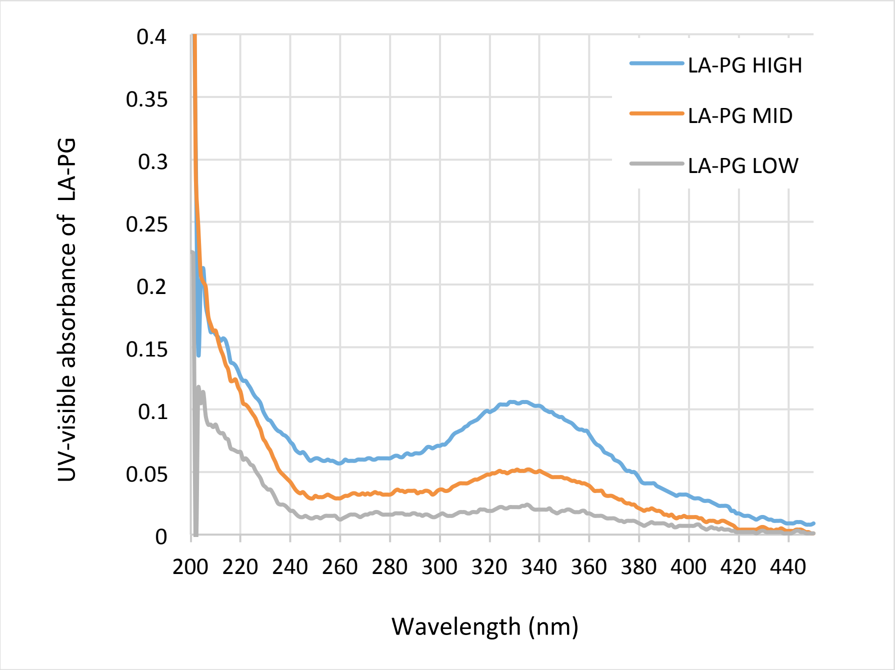

Fig. S2. UV-visible single spectrum of LA-PG showing three levels of BHHTEGST conjugation with PG. The band at 335 nm (from BHHTEGST component absorbance) intensifies with respect to the band at 280 nm (from protein component absorbance) as a result of the number of attached equivalents of BHHTEGST is increased. Absorption at 320 nm was used for quantification of number of BHHTEGST per PG.

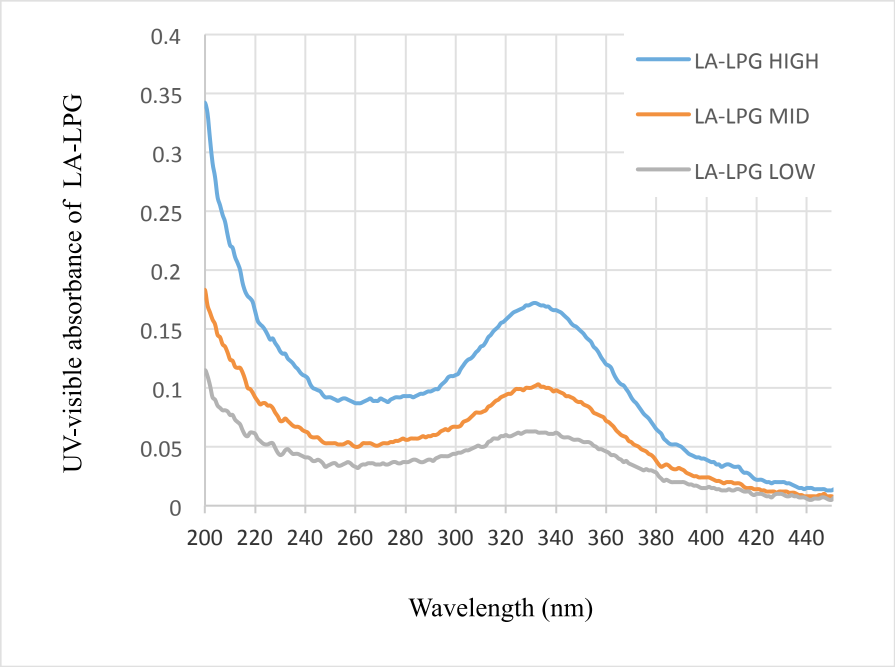

Fig. S3. UV-visible single spectrum of LA-LPG showing three levels of BHHTEGST conjugation with LPG. The band at 335 nm (from BHHTEGST component absorbance) intensifies with respect to the band at 280 nm (from protein component absorbance) as a result of the increasing number of attached equivalents of BHHTEGST. Absorption at 320 nm was used for quantification of number of BHHTEGST per LPG.
